# Combination of sorafenib and TACE inhibits portal vein invasion for intermediate stage HCC: a single center retrospective controlled study

**DOI:** 10.18632/oncotarget.20745

**Published:** 2017-09-08

**Authors:** Qi Yao, Hongsen Zhang, Bin Xiong, Chuansheng Zheng

**Affiliations:** ^1^ Department of Radiology, Union Hospital, Tongji Medical College, Huazhong University of Science and Technology, Hubei, China

**Keywords:** sorafenib, hepatocellular carcinoma (HCC), transcatheter arterial chemoembolization (TACE), portal vein invasion, combined therapy

## Abstract

**Purpose:**

This study aims to investigate the effect of sorafenib plus Transarterial Chemoembolization (TACE) treatment on inhibiting portal vein invasion in patients with intermediate stage HCC.

**Materials and Methods:**

The consecutive medical records of patients with HCC were retrospectively analyzed from October 2009 to February 2015. The propensity score matching method was applied into group matching. The Kaplan-Meier method and the Log-Rank Test was used to estimate the median survival time, median time to progression and median time to portal vein invasion. Factors associated with survival benefits were identified by univariate and multivariate Cox-regression model analyses.

**Results:**

Of 97 patients enrolled, 19 patients received TACE-sorafenib treatment and 78 patients received TACE treatment. During the follow-up period of 15 months, the median time to portal vein invasion was 14.2 months vs 8.77 months, respectively (p=0.073). And the analysis of the cox's proportional hazard model revealed that patients treated with TACE treatment alone would run greater risk of portal vein invasion compared with TACE-sorafenib treatment (hr=7.49, p=0.021). Early administration of sorafenib was associated with lower risk of portal vein invasion (p=0.021) according to the univariate analysis. Adverse events (AEs) identified in the combined group were mostly classified as Grades 1 and 2, and skin-related reactions and fatigue were the most common.

**Conclusions:**

Sorafenib may could inhibit portal vein invasion of hepatoma carcinoma cells. Early administration of sorafenib may bring more survival benefits.

## INTRODUCTION

Hepatocellular carcinoma (HCC) is the third leading cause of cancer-related death and is the fifth most common cancer worldwide [1]. Despite the improving diagnostic technologies nowadays, there remains large number of patients are already progressed at the time of diagnosis and are not candidates for curative treatment like surgical resection and liver transplantation [2–4]. As it is impossible to perform curative therapies, patients have to rely on non-surgical therapies such as chemotherapy, transcatheter arterial chemoembolization or embolization(TACE or TAE), radiotherapy, targeted therapy, or immunotherapy to prolong their survival time [5–8]. Transarterial chemoembolization(TACE), recognized as an effective palliative treatment option for patients with advanced HCC, is now widely performed for unresectable liver cancer which classified as intermediate stage according to the Barcelona Clinic Liver Cancer(BCLC) staging system [2, 9–11]. However, in many cases tumor cells can adapt to the highly anaerobic microenvironment resulted from TACE through the negative feed-back response and incomplete embolization, which often correlated with the recurrence and metastasis of tumor.

Sorafenib which has been reported can prolong the overall survival(OS) of HCC patients, is now recommended as the firstline therapy for systemic treatment of HCC [12, 13]. This molecular targeted agent can inhibit the proliferation of tumor cells by inhibiting the Raf serine/threonine kinases as well as inhibit tumor angiogenesis by inhibiting the receptor tyrosine kinase activity of Vascular Endothelial Growth Factor(VEGFF) receptors (VEGFRs) 2 and 3, which lays the theoretical foundation of combining TACE therapy and sorafenib together [14–17]. Most recently, more and more researches which focused on the combination of TACE and sorafenib have also showed optimal results in term of overall survival.

Portal vein tumor thrombus (PVTT) is an important cause of intrahepatic relapse and metastasis of HCC. It is reported that PVTT occurs in up to 44% of patients with HCC and approximately 10%–40% of patients had portal vein invasion when diagnosed with HCC [18–20]. The occurrence of PVTT limits the application of surgery and interventional therapy which seriously affect the prognosis of patients with HCC. Once ocurred with PVTT, their survival time tend to be greatly reduced [19–21]. Hence, an early inhibition of vascular invasion which help preventing the formation of PVTT is necessary for prolonging the survival time of HCC patients.Recently, a growing body of researches have reported that sorafenib brings survival benefits to patients with HCC and PVTT in the first- or lower-order portal vein branches [22–24]. In our clinical work, we also found that the occurrence of PVTT becomes rare in patients who received TACE-sorafenib treatment. And even when occurred with PVTT, the type of it would be second- or lower-order branches.

Hence, we hypothesized that sorafenib could inhibit or reduce portal vein invasion of hepatoma carcinoma cells and thus guarantee the perfusion of the liver in a period, and eventually bring survival benefit to HCC patients. Therefore, based on our hypothesis, we conducted this retrospective study to compare the condition of portal vein invasion of patients received TACE-sorafenib treatment versus TACE therapy alone. The OS and TTP of the two groups were evaluated as well.

## RESULTS

**Figure 1:**
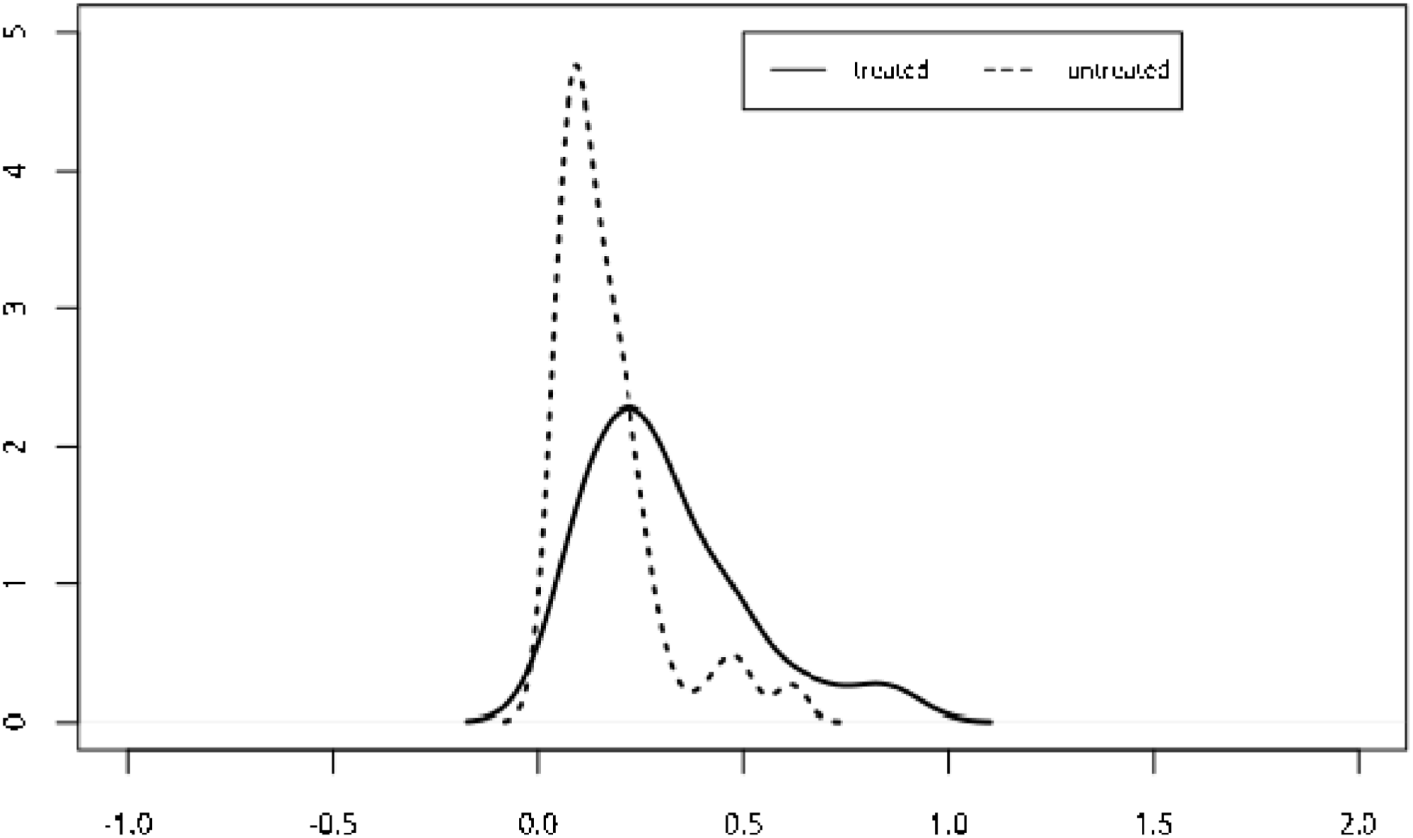
Propensity score matching was used and was included as independent variable into a COX model, and no significant difference was showed in baseline data (p=0. 382, hr=1.55)

Propensity score matching method was used into group matching, and eventually 19 cases were enrolled into the TAice TACE procedures. Baseline data (Table 1) of the two groups didn’t show significant differences when applied with propensity score matching method (Figure 1). And the follow-up period ranged from 2 months to 56 months and the median follow-up period was 33 months. Of which, the follow up period of the combined group ranged from 3 months to 49 months and the median follow-up period is 25.5 months, while the period of the mono therapy ranged from 2 months to 56 months and the median of which was 14.3 months. The most frequently observed AE was skin related reaction (63.2% patients with hand–foot reaction and 5.3% with rash) followed by fatigue in 52.6%, diarrhea in 36.8%, hypertension in 34.3%, and mouth ulceration in 21.2%, muscle aches in 10.6%. However, the majority of AEs were classified as Grades 1 and 2, suggesting the relative safety of this combination therapy.

**Table 1:** Baseline data of the patients included

Variables	TACE (n=78)	Sorafenib&TACE (n=19)	P value
Gender			
Male	78	19	
Female	0	0	
Age (years)	46.67±8.5	45.32±5.94	0.62
Tumor Size (mm)	6.23±3.86	5.67±3.54	0.55
Number of TACE Procedure	3.95±2.02	5.89±2.26	0.002
Under Surgery (before TACE Treatment)			0.05
NO	67	12	
YES	11	7	
BCLC Stage			1
A	18	5	
B	60	14	
Child-Pugh Class			0.58
A	63	15	
B	15	4	
AFP			1
≤100ng/ml	14	3	
>100ng/ml	64	16	
Cause of Liver disease			0.72
Hepatitis B or C	75	18	
Other	3	1	

### The condition of portal vein invasion

**Figure 2:**
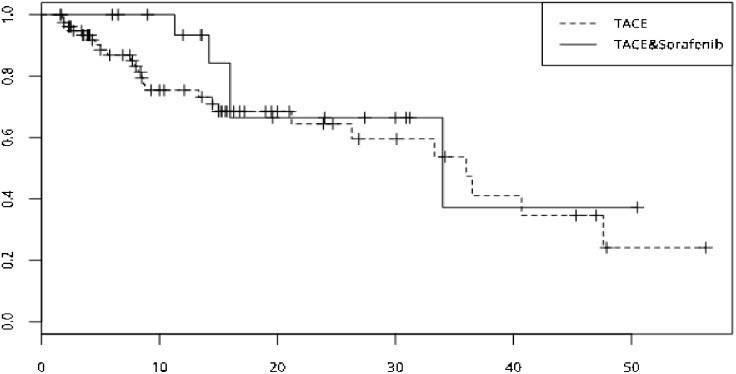
Survival curve of the two group was drawed by Kaplan-Meier method, median time to portal vein invasion of the two group showed no significant differences (TACE-sorafenib vs TACE, 34 months vs 36 months, p=0. 327)

**Figure 3:**
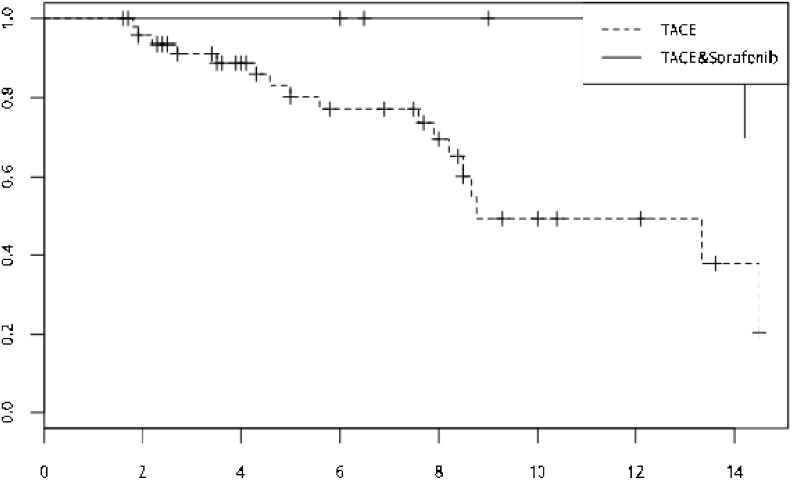
During the follow-up period of 15 months, after propensity score matching, the hazard of portal vein invasion of the TACE monotherapy was higher than the TACE-sorafenib group (hr=7.49, p=0.021)

Until the endpoint, 30 cases of all the patients included ihad been developed with PVTT of different type. Of which, the combined group 5 cases, the mono group 25 cases. Median time to portal vein invasion showed no significant differences (the Combined Group vs the TACE group34months vs 36months, respectively, p=0.327), and survival curves were calculated for both groups by using Kaplan-Meier methods (Figure 2). Based on survival curve, we found that within the follow-up period of 15 months, the survival of the comibined group was much better than the TACE group, so we set 15 months as the cutting point and further evaluated the portal vein conditions within the follow-up period of 15 months. And the results showed that during the follow-up period of 15 months, 19 cases of all patients occured with PVTT. Of which 2 cases in the combined group and 17 cases in the mono group. And the analysis of the primary endpoint showed no significant differences of the two groups in term of the median time of portal vein invasion( the Combined Group vs the TACE group, 8.77months vs 14.2months, respectively, p=0.073). The analysis of the cox's proportional hazard model revealed patients with TACE-sorafenib treatment may have a lower hazard of portal vein invasion (the TACE Group vs the Combined Group, hr=7.49, p=0.021) (Figure 3).

### Survival

**Figure 4:**
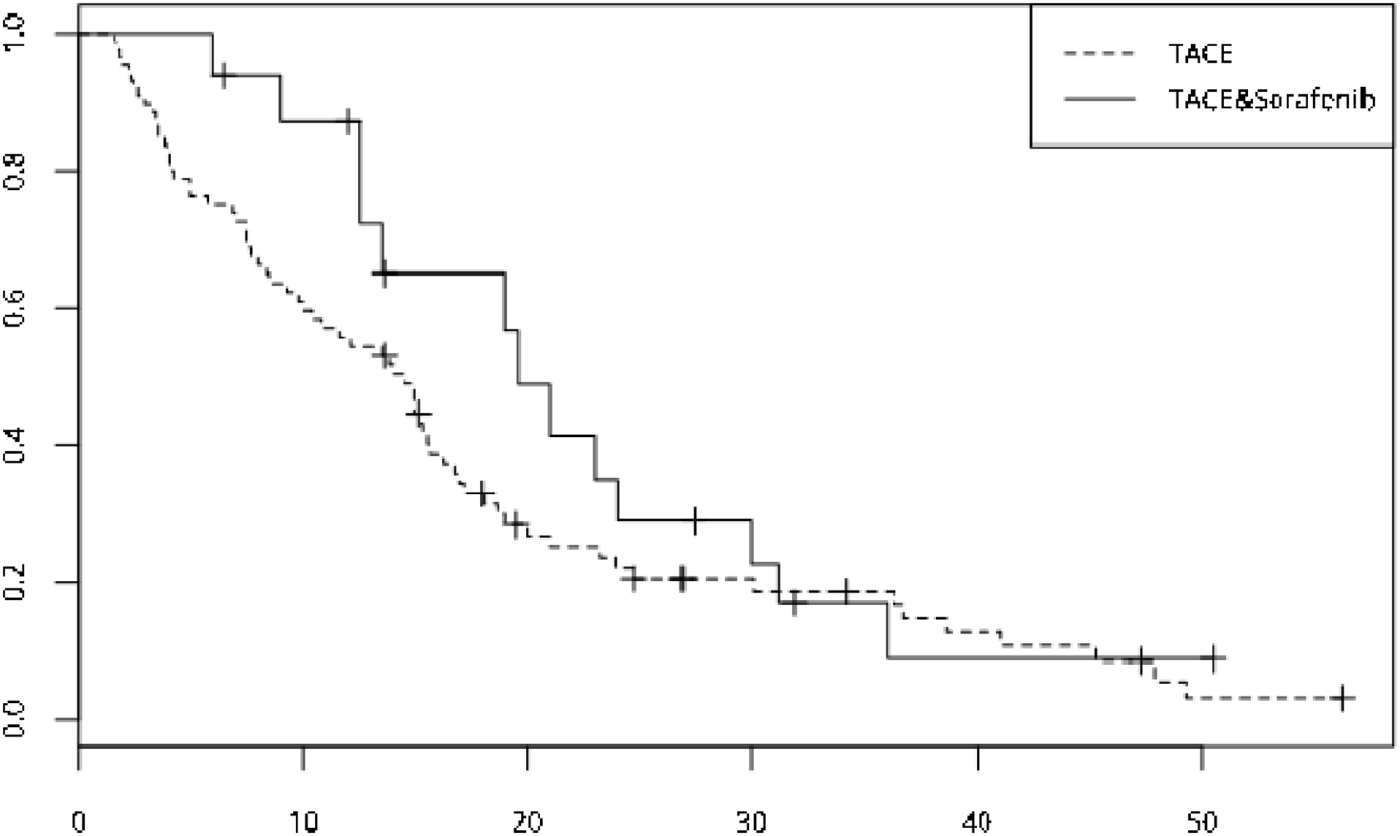
The OS curve of the two group--the TACE plus sorafenib group, n=19, median OS is 23 months, the TACE group, n=78, median OS is 13.8 months and no significant differences was showed (p=0.061)

Until the endpoint of observation, 81 cases of all patients had been died or lost follow-up, and of which 13 cases in the TACE-sorafenib group, while 68 cases in the TACE group. The analysis of the OS showed mdian OS of the two groups showed no significant differences(the Combined Group vs the TACE group, 23.0 months vs 8.8 months, respectively, p=0.061) (Figure 4).

### Time to progress

**Figure 5:**
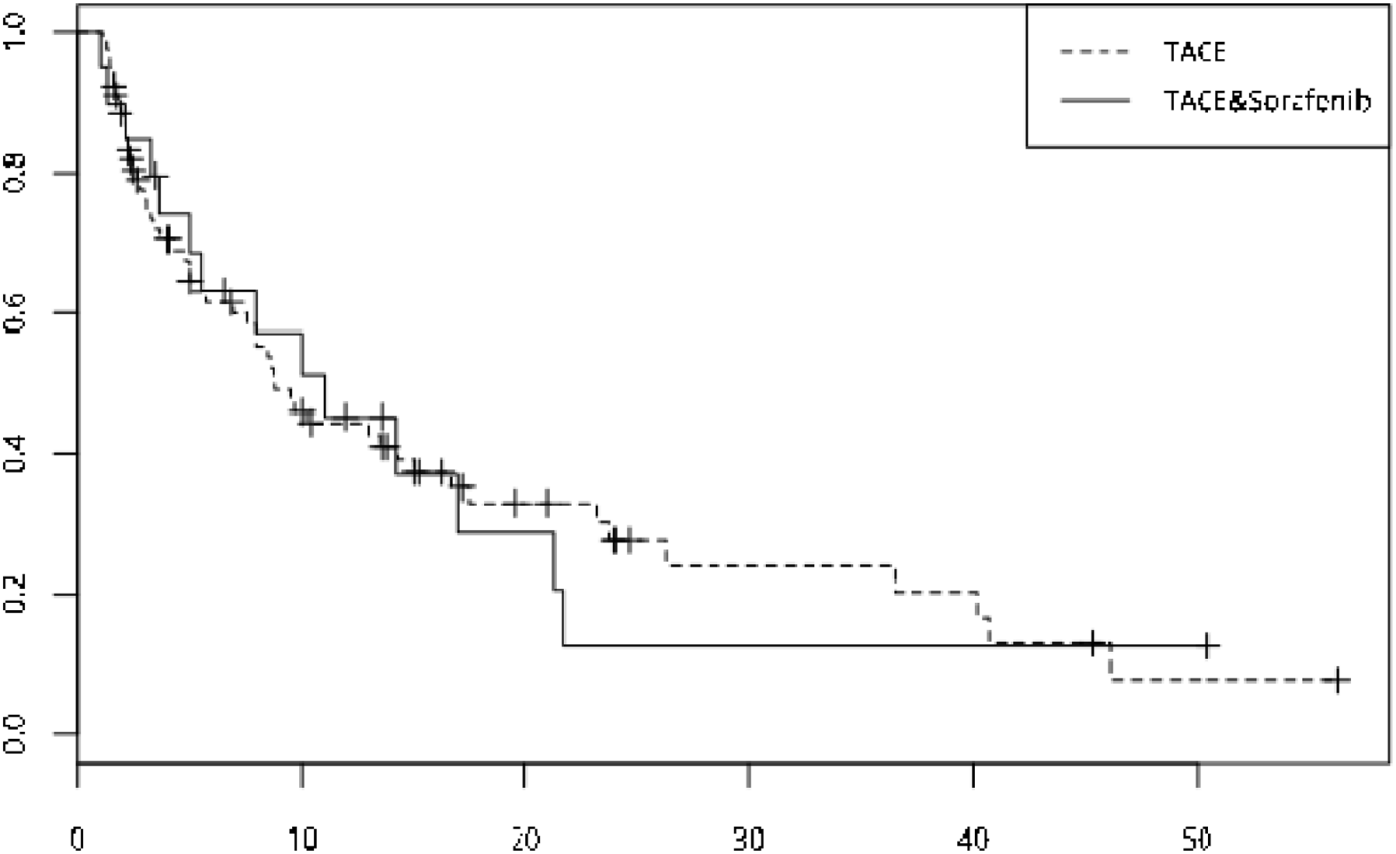
TTP curve of the two group-- the median TTP showed no significant differences (TACE-sorafenib vs TACE, 10 months vs 8.8 months, p=0.061)

Until the endpoint, 66 cases of all patients had been progressed, of which 14 cases in the TACE-sorafenib group, 52 cases in the TACE group. Disease progression was defined in accordance with the modified Response Evaluation Criteria in Solid Tumors (mRECIST). The log rank test revealed that the median TTP of the combined group wasn’t significantly longer than the TACE group(the Combined Group vs the TACE Group, 10.0months vs 8.8months, respectively, p=0.829) (Figure 5).

### Univariate analysis

**Figure 6:**
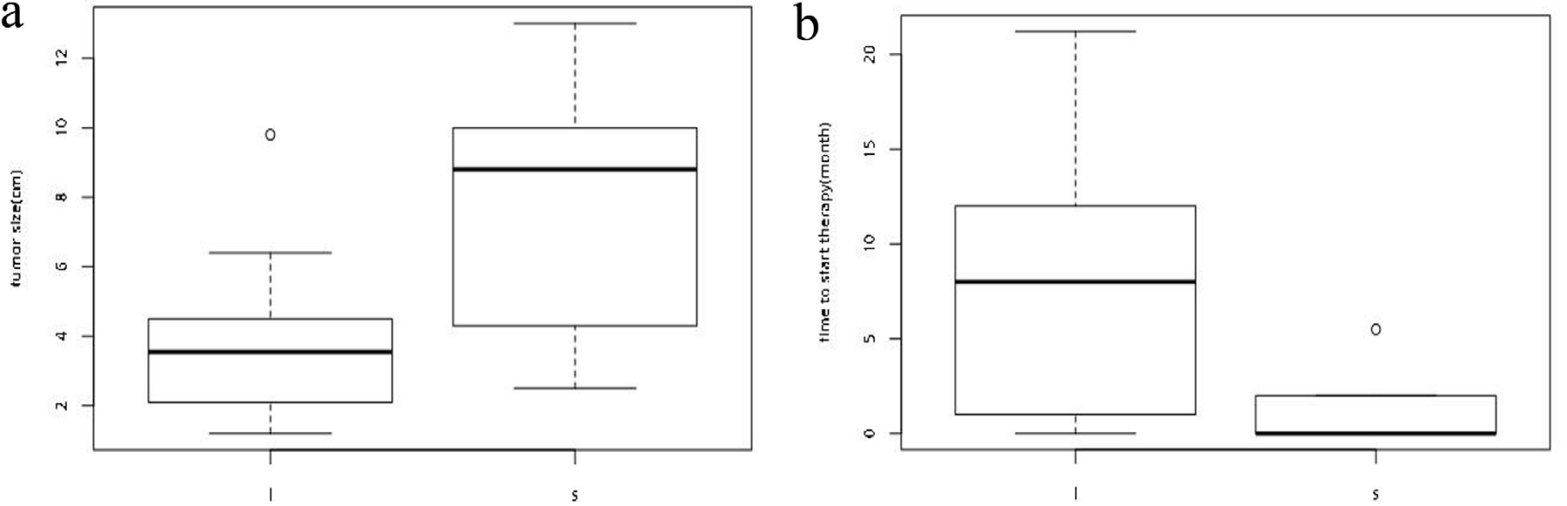
Univariate analyses revealed that the early administation of sorafenib was significantly associated with better OS—**(a)**, the box-plot of tumor size (p=0. 027); **(b)**, the box-plot of time to start administration of sorafenib (p=0.021).

Univariate analysis was also performed in those patients of which the follow-up period were within 15 months. And the analysis revealed that tumor size (Figure 6a) (p=0.027) and the time to start sorafenib administration (Figure 6b) (p=0.021) was significantly associated with OS. Moreover, the Box-plot revealed that the tumor sizes of patients who took early administration of sorafenib was even bigger than those who began the therapy later which signified that an early administation of sorafenib may bring more survival benefit to HCC patients.

## DISCUSSION

Portal vein tumor thrombus (PVTT) is an important cause of intra hepatic relapse and metastasis of HCC. The occurrence of PVTT limits the application of surgery and interventional therapy which seriously affect the prognosis of patients with HCC. Recently, a growing body of literature have highlights the effect of TACE-sorafenib treatment on prolonging the overall survival to HCC patients with PVTT [22–24]. A restropective study had compared the effect of TACE-sorafenib treatment with TACE mono-therapy for the patients of HCC with PVTT [22]. The study revealed that compared with TACE treatment, TACE-sorafenib therapy could prolong the survival time of patients with advanced HCC who developed a tumor thrombus in the first or lower-order portal vein branches (OS 11months vs 6 months, TACE-sorafenib vs TACE, p<0.01, respectively). And they concluded that TACE-sorafenib yielded a promising outcome in patients with advanced HCC who developed a tumor thrombus in the first or lower-order portal vein branches. Two other retrospective studies which also studied this combined treatment on patients occured with PVTT had showed similar results. However, despite the emerging treatment strategies, once occured with PVTT, the survival time of HCC patients tended to be greatly reduced. Therefore, inhibiting microvascular invasion at an early stage, preventing the formation of PVTT and guaranteeing the perfusion of liver is necessary for prolonging the survival time of HCC patients. But so far, there is no such research which emphasized on investigating the effect of the current treatment methods on the inhibition of PVTT.

In the past decades, a growing body of researches have highlights the treatment effect of combining sorafenib with TACE therapy.Theoretically, sorafenib combined with TACE therapy could reduce the level of vEGF and other factors which was often overexpressed by the hypoxia microenviroment induced by repeated TACE procedures. At present, clinical trials which put emphasis on such combined treatment s revealed that TACE-sorafenib treatment could prolong the survival time of advanced HCC patients. Choi etc reported that TACE-sorafenib treatment yielded a promising outcome in prolonging OS and TTP in patients with advanced HCC [25]. Pawlik et al reported that sorafenib combined with TACE showed a promising disease control rate of 95% according to RESIST criteria with manageable toxicity [26]. All those results revealed that the combining therapy showed a promising effect on HCC patients than TACE alone or sorafenib alone. However, despite all those promising results, when faced with liver cancer, all the present treatment showed marginal effect, thus, more clinical trials are needed to optimize treatment strategies.

Hence, based on above, in this present study, we tried to investigate whether sorafenib has the inhibitory effect of tumor metastasis, and thus, we for the first time investigated the effect of combing TACE with sorafenib on inhibiting the vascular invasion in HCC patients through this retrospective research. And in order to assess such effect of sorafenib, we for the first the time set the time to portal vein invasion as the primary endpoint of our research, while overall survival and the time to progress was set as the seconary goal.

And eventually, within the follow-up period of 15 months, 19 cases of all patients occured with PVTT, of which 2 cases in the combined group, 17 cases in the mono-group, median time to portal vein invasion didn’t show significant differences (the Combined Group vs the TACE Group, 8.77months vs 14.2months, respectively, p=0.073). However, The analysis of the cox's proportional hazard model revealed that compared with TACE mono-therapy, patients who recieved TACE-sorafenib treatment may have a lower hazard of portal vein invasion.(hr=7.49, TACE vs TACE-sorafenib, p=0.021). This results revealed that sorafenib may has an inhibitory effect on tumor metastasis from the perspective of pathology, orafenib could inhibit effect This may explain the mechanisms of the anti-tumor effect of sorafenib from the perspective of pathology, namely, sorafenib could inhibit or reduce portal vein invasion of hepatoma carcinoma cells which in turn brings survival benefit to HCC patients. Furthermore, analysis of the AEs experienced by patients receiving TACE-sorafenib treatment showed that most were low-grade, suggesting that this combination treatment strategy is both efficacious and safe.

Moreover, based on the results we investigated above, we perfomed the univariate analysis with rank-sum test and Fisher exact test to investigate the related factors associated with survival benefits in those patients of which the follow-up period were within 15 months. And eventually, the analysis revealed that the tumor size and the time of starting sorafenib administration are the two risk factors which affect the prognosis of patients with HCC. Furthermore, in our study, the size of tumor in the combined group is much bigger than the mono-group while the hazard of portal vein invasion was reduced in the combined group, thus, we concluded that early administation of sorafenib may bring more survival benefit, which further confirmed what R. Sacco, A. Romano, M. Bertini had reported earlier—HCC patients starting sorafenib after one single TACE procedure present improved OS and disease control rate (DCR) with respect to those who received ≥2 TACE procedures [27]. Moreover, another study which enrolled 192 cases of HCC patients who start sorafenib after one single TACE procedure showed that Median progression-free survival and time to progression were 384 and 415 days, respectively, and the estimated 3-year overall survival was 86.1%, which on one hand reflected that early adminisration may bring more survival benefit to HCC patients [28]. Hence, we concluded that early administration of sorafenib would be recommended to HCC patients.

Several limitations exist in this present study. We failed to reveal that TACE-sorafenib therapy could prolong the survival time of HCC patients as some literature reported before. This unsatistactory results may owing to that when assess the endpoint of TTP, the time to portal vein invasion wasn’t singlely evaluated while the intrahepatic relapse and extrahepatic metastasis are also included. Secondly, most of the patients included in the combined group started taking sorafenib when disease progressed as metastasis or relapse. But at the same time, we also found that, of the 19 cases included in the TACE-sorafenib group, only 1 case of HCC patients had witnessed the development of PVTT. And in the other 18 cases, portal vein invasion had occured after relapse or metastasis. And moreover, of which 12 cases had not developed with PVTT till death or lost follow up. And in those patients who developed with PVTT, the tumor thrombus was developed in the second or lower-order portal vein branches. This may help confirm what T. Pa n, *et al* reported before that combined therapy is highly effective in stabilizing PVTT, which prevents further portal vein obstruction and hepatic function deterioration [24]. Furthermore, our study didn’t show significant differences in OS between the two groups. And this may owing to that the sample size of the combined group is too small as well as the whole condition of tumor was more worse than the mono-group. Also, the time of sorafenib administration was not that strict in the combined group may also account for this depressing results.

In conclusion, through this retrospective study, we concluded that sorafenib could inhibit portal vein invasion of hepatoma carcinoma cells. And early administration of sorafenib which may bring more survival benefit is recommended to HCC patients. However, further prospective randomized trials are required to confirm these observations.

## MATERIALS AND METHODS

### Patients

The consecutive medical records of HCC patients in our hospital from October 2009 to December 2015 were retrospectively reviewed by two senior doctors of high qualification. Ultimately, a total of 116 cases were enrolled, among which 19 cases received TACE-sorafenib treatment and 98 cases recieved TACE treatment alone. The treatment strategies were made according to the patients' wishes and the recommendation of the attending physicians. The propensity score matching method was used into group matching, and eventually 19 cases were enrolled into the TACE-sorafenib group while 78 cases were enrolled into the TACE group. The inclusion criteria were: (1) HCC was confirmed by pathological examination or according to the European Association for the Study of the Liver(EASL) criteria; (2) No portal vein invasion was observed through enhanced CT or MRI imaging before starting the treatment procedures; (3) The stage of HCC was classified as intermediate stage according to the BCLC system; (4) Eastern Cooperative Group performance status (ECOG) score of 0–2; (5) Child-Pugh class A or B. And the exclusion criteria were defined as follows: (1) Patients received palliative operative liver transplantation or radiotherapy during the period of treatment; (2) Patients suffered from another type of carcinoma in addition to HCC (Table 2); (3) Hepatic encephalopathy, severe ascites, oesophageal or gastric fundal variceal bleeding or other serious medical comorbidities. Informed consent were signed before therapy.

**Table 2:** The flow chart of patients included

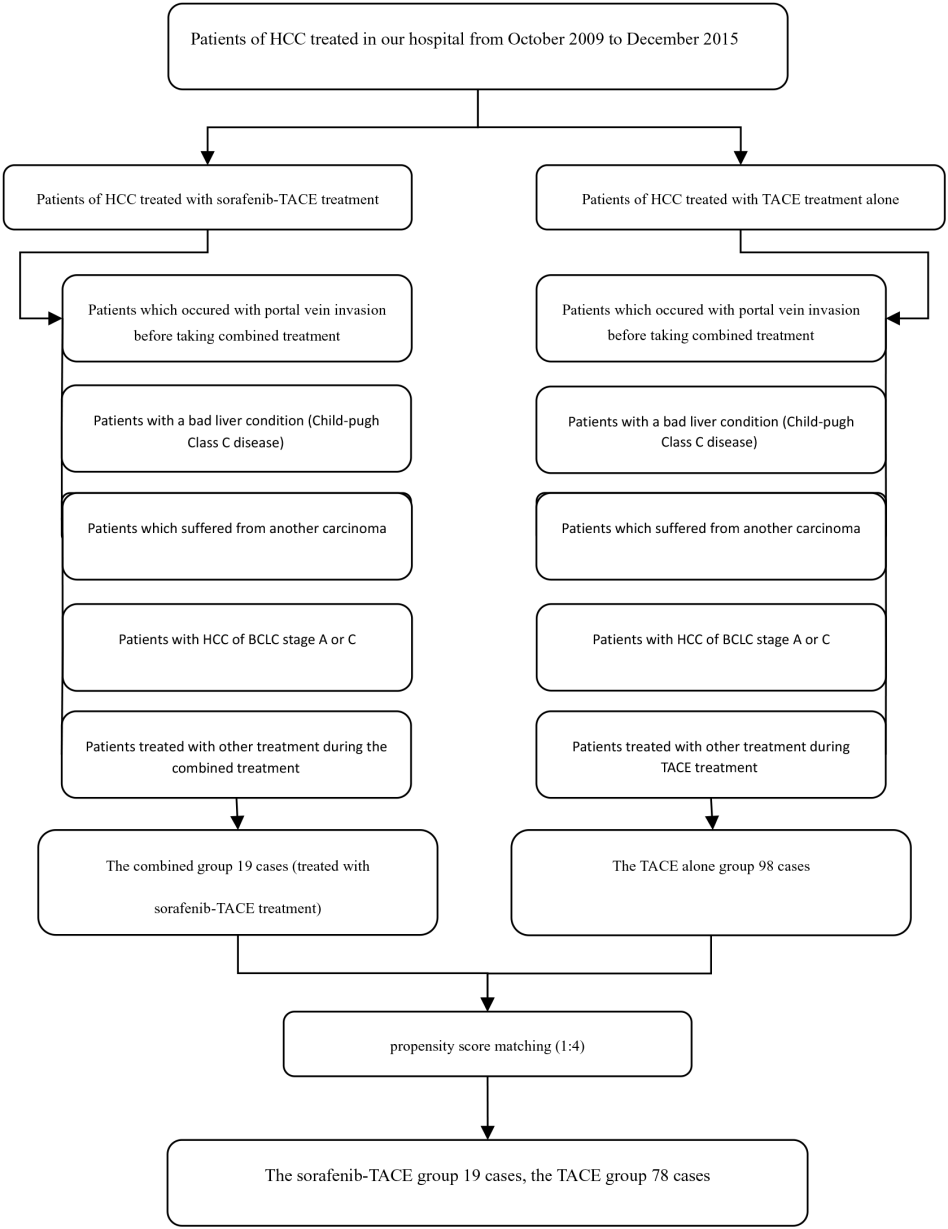

### TACE procedure

TACE was performed with digital subtraction angiography (Siemens, Artbt Zee, Germany). A 5-F Yashiro catheter (Terum, Japan) or R-H catheter (Cook, USA) or microcatheter (Progreat, Terumo, Tokyo, Japan) was inserted into the hepatic artery through the femoral artery using the Seldinger technique. And then make sure the size, number and location of the tumor and then assess the arterial blood supply of the liver and identify the accessory arteries, a superior mesenteric artery angiography was conducted when necessary. A superselective catheterization of the tumor-feeding artery was conducted by the 2.7F Progreat microcatheter. An emulsion of 2-20 ml of lipiodol (Laboratorie Guerbet, Aulnay-sous-Bois, France), 20-60 mg of epirubicin (Farmorubicin; Pharmacia, Tokyo, Japan), and followed by embolization with gelatin sponge particles dissected by an operator into 2-3 mm diameter pieces (Gelfoam; Hangzhou, China). The procedure was performed according to the experience gained from previous work.(19) The dose of that can be adjusted depending on the size, location, arterial supply of the tumor and the condition of the hepatic function.

### Sorafenib administration

Sorafenib (Nexavar; Bayer, Leverkusen, Germany) was taken at a dose of 400mg twice a day (400, g, bid), and adverse effect was accessed according to the National Cancer Institute Common Toxicity Criteria for Adverse Events, NCI-CTCAE, version 3.0, if serious adverse event(NCI-CTCAE 3-4 grade) was observed, then dose of it would be reduced to 200mg twice a day or temporarily discontinued until toxicity was decreased. The administration time of sorafenib was until death or serious adverse events occurred. During the treatment period, the administration of sorafenib should be continued if the status of tumor was evaluated as progressed.

### Follow-up

Regular follow-up was conducted for those patients which enrolled into this retrospective study. Enhanced CT or MRI was performed every 4-6 weeks and relevant laboratory examination (Liver function, AFP level, blood coagulation function, etc) was conducted as well. If residual lesions or introhepatic relapse was revealed by enhanced CT or MRI images, then repeated TACE procedure would be performed.

### Study endpoints

The primary goal was the time to portal vein invasion, while OS and TTP was set as the secondary endpoint. The time to portal vein invasion was defined as the time from the enrollment to the time that the patient was confirmed with PVTT by enhanced CT/MRI imaging. And OS was defined as the time from enrollment to death of any causes or to the last follow-up in censored patients, while TTP was defined as the time from the start of the first TACE procedure until tumor progression. Tumor progression was based on the modified Response Evaluation Criteria in Solid Tumors (mRECIST) which include intrahepatic relapse, lymph node metastasis and extrahepatic metastasis, PVTT, etc.

### Statistical analysis

All data was analyzed by using R language version 3.1.0, and P<0.05 indicated a significant differences. Propensity score matching was used into group matching, and the Kaplan-Meier method was used to estimate the median survival time, median time to progression and time to portal vein invasion. Survival curves were calculated for both groups by using Kaplan-Meier methods and to determine significant differences between the median survival time of the two groups, the Log-Rank Test was used. To evaluate the related factors of survival benefit in the TACE-sorafenib Group, univariate analyses were performed with rank-sum test and Fisher exact tests.

## Abbreviations

TACE: Transcatheter Arterial Chemoembolization
HCC: Hepatocellular Carcinoma
BCLC: Barcelona Clinic Liver Cancer
OS: Overall Survival
TTP: Time to Progress
VegF: Vascular Endothelial Growth Factor
VegFRs: Vascular Endothelial Growth Factor Receptors
PVTT: Portal Vein Tumor Thrombus
EASL: European Association for the Study of the Liver
mRECIST: the Modified Response Evaluation Criteria in Solid Tumors
